# Supplementary material for: Temporal Trends in Oral Anticoagulant Prescription in Atrial Fibrillation Patients between 2004 and 2019
Source: Int J Environ Res Public Health. 2022 May 4;19(9):5584. doi: 10.3390/ijerph19095584 (PMC9101720; doi:10.3390/ijerph19095584)
Supplement: Supplementary file 1 [file ijerph-19-05584-s001.zip › trends fig S2.pdf]

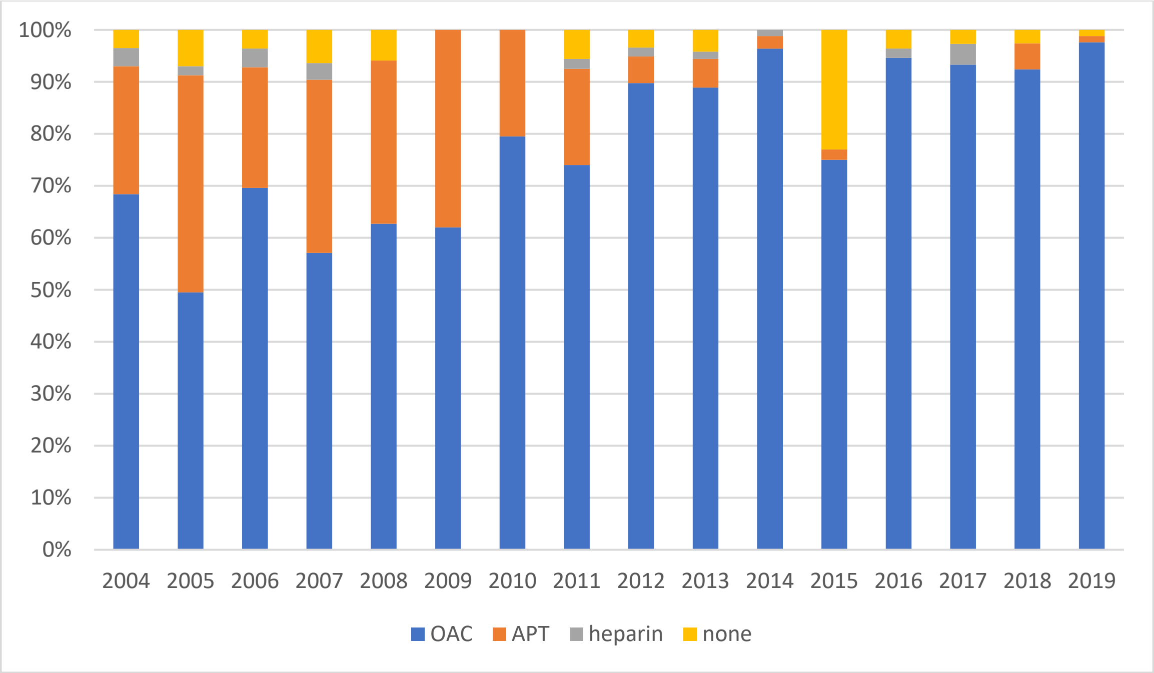

Figure S2. Temporal trends of antithrombotic therapy in intermediate stroke risk patients. Abbreviation: APT, antiplatelet drug; OAC, oral anticoagulant.
