# Supplementary material for: Temporal Trends in Oral Anticoagulant Prescription in Atrial Fibrillation Patients between 2004 and 2019
Source: Int J Environ Res Public Health. 2022 May 4;19(9):5584. doi: 10.3390/ijerph19095584 (PMC9101720; doi:10.3390/ijerph19095584)
Supplement: Supplementary file 1 [file ijerph-19-05584-s001.zip › trends tab S1.pdf]

Table S1. Risk factors in the CHA_2DS_2-VASc score and in the HAS-BLED score [5].

Clinical characteristic	Definition
**CHA_2DS_2-VASc score**	
Heart failure	clinical heart failure, or objective evidence of moderate to severe left ventricular dysfunction, or hypertrophic cardiomyopathy
Hypertension	hypertension or on antihypertensive therapy
Stroke	previous stroke, transient ischaemic attack, or thromboembolism
Diabetes mellitus	treatment with oral hypoglycaemic drugs and/or insulin or fasting blood glucose >125 mg/dL (7 mmol/L)
Vascular disease	angiographically significant coronary artery disease , previous myocardial infarction, peripheral artery disease, or aortic plaque
Sex category	female
Age ≥ 75, 64-74	-
**HAS-BLED score**	
Uncontrolled hypertension	SBP >160 mmHg
Abnormal renal and/or hepatic function	dialysis, transplant, serum creatinine >200 mmol/L, cirrhosis, bilirubin ≥2 upper limit of normal, AST/ALT/ALP ≥3upper limit of normal
stroke	previous ischaemic or haemorrhagica stroke
bleeding history or predisposition	previous major haemorrhage or anaemia or severe thrombocytopenia
labile INR	TTR <60% in patient receiving VKA
elderly	aged >65 years or extreme frailty
drugs or excessive alcohol drinking	Concomitant use of antiplatelet or NSAID; and/or excessivec alcohol per week (cAlcohol excess or abuse refers to a high intake (e.g. >14 units per week), where the clinician assesses there would be an impact on health or bleeding risk)

Abbreviation: ALP, alkaline phosphatase; ALT, alanine aminotransferase; AST, aspartate aminotransferase; SBP, systolic blood pressure; INR, international normalized ratio; NSAID, Non-steroidal anti-inflammatory drug; TTR, time in therapeutic range; VKA, vitamin K antagonist. CHA_2DS_2-VASc score: congestive heart failure (1 point), hypertension (1 point), age ≥ 75 years (2 points), diabetes mellitus (1 point), stroke/TIA/thromboembolism (2 points), vascular disease (1 point), age 65–74 years (1 point), sex female (1 point). HAS-BLED score: hypertension (1 point), liver disease (1 point), renal disease (1 point), stroke history (1 point), bleeding history (1 point), age >65 years (1 point) and drug (concomitant use of NSAID or antiplatelet agent, 1 point).
