# Supplementary material for: Temporal Trends in Oral Anticoagulant Prescription in Atrial Fibrillation Patients between 2004 and 2019
Source: Int J Environ Res Public Health. 2022 May 4;19(9):5584. doi: 10.3390/ijerph19095584 (PMC9101720; doi:10.3390/ijerph19095584)
Supplement: Supplementary file 1 [file ijerph-19-05584-s001.zip › trends tab S2.pdf]

Table S2. Baseline characteristic of study group according to stroke prevention.

Clinical characteristic	High stroke risk n=8264	Intermediate stroke risk n=1024	Low strok risk n=368
**Type of atrial fibrillation**			
Paroxysmal	3650 (44.3)	514 (50.2)	225 (61.1)
Persistent	1022 (12.4)	305 (29.8)	112 (30.4)
Permanent	3592 (43.5)	205 (20)	31 (8.4)
**Stroke risk factors**			
Age, years<br>Mean (SD)<br>Median (IQR)	 73.7 (9.4)<br>74 (67-81)	 58 (8.5)<br>59 (54-63)	 51.4 (10.4)<br>54 (46-59)
<65	1257 (15.2)	862 (84.2)	368 (100)
65-74	2907 (35.2)	162 (15.8)	0
≥ 75	4100 (49.6)	0	0
Female	3790 (35.2)	337 (32.9)	94 (25.5)
Heart failure	5409 (65.5)	258 (5.2)	0
Hypertension	6837 (82.7)	550 (53.7)	0
Previous stroke/ TIA/peripheral embolism	1266 (15.3)	0	0
Diabetes mellitus	2448 (29.6)	15 (1.5)	0
Vascular disease	3325 (40.2)	39 (3.8)	0
**Medical history**			
Bleeding	247 (3)	13 (1.3)	7 (1.9)
Cancer	369 (4.5)	27 (2.6)	12 (3.3)
Peptic ulcer disease	281 (3.4)	30 (2.9)	15 (4.1)
Anemia	1538 (18.6)	73 (7.1)	18 (4.9)
Thrombocytpenia	1319 (16)	114 (11.1)	24 (6.5)
eGFR < 60 ml/min/1,73m2	5525 (66.9)	414 (40.4)	104 (28.3)
**Bleeding risk**			
HAS-BLED<br>Mean (SD)<br>Median (IQR)	 2 (0.8)<br>2 (2-2)	 0.8 (0.5)<br>1 (0-1)	 0.06 (0.2)<br>0 (0-0)
HAS-BLED ≥ 3	1675 (20.3)	2 (0.2)	0
**Reason for hospitalization**			
CIED implantation /reimplantation	2064 (25)	108 (10.5)	35 (9.5)
Heart failure	1879 (22.7)	109 (10.6)	10 (2.7)
Acute coronary syndrome/planned PCI	1109 (13.4)	92 (9)	12 (3.3)
AF without any procedures	810 (9.8)	214 (20.9)	98 (26.6)
Electrical cardioversion	711 (8.6)	243 (23.7)	102 (27.7)
Ablation	204 (2.5)	83 (8.1)	52 (14.1)
Other	1487 (18)	175 (17.1)	59 (16)
**Years of hospitalization**			
2004-2006	1172 (14.2)	168 (16.4)	65 (17.7)
2007-2010	1387 (16.8)	208 (20.3)	68 (18.5)
2011-2012	878 (10.6)	113 (11)	40 (10.9)
2013-2016	2348 (28.4)	258 (25.2)	91 (24.7)
2017-2019	2479 (30)	277 (27.1)	104 (28.3)

The numbers are presented as the mean (standard deviation), median (interquartile range), or numbers (percentage) otherwise mentioned. Abbreviation: AF, atrial fibrillation; CIED, cardiac implantable

electronic device; IQR, interquartile range; SD, standard deviation; TIA, transient ischemic attack. CHA_2DS_2-VASc score: congestive heart failure (1 point), hypertension (1 point), age ≥ 75 years (2 points), diabetes mellitus (1 point), stroke/TIA/thromboembolism (2 points), vascular disease (1 point), age 65–74 years (1 point), sex female (1 point). HAS-BLED score: hypertension (1 point), liver disease (1 point), renal disease (1 point), stroke history (1 point), bleeding history (1 point), age >65 years (1 point) and drug (concomitant use of NSAID or antiplatelet agent, 1 point).
